# A network meta-analysis on the effectiveness and safety of acupuncture in treating patients with major depressive disorder

**DOI:** 10.1038/s41598-021-88263-y

**Published:** 2021-05-17

**Authors:** Hu Zhichao, Lam Wai Ching, Li Huijuan, Yao Liang, Wang Zhiyu, Huang Weiyang, Bian Zhaoxiang, Zhong L. D. Linda

**Affiliations:** 1 Hong Kong Chinese Medicine Clinical Study Center, School of Chinese Medicine, Hong Kong Baptist University, Kowloon, 999077 Hong Kong SAR China; 2 School of Public Health, Lanzhou University, Lanzhou, 730000 China; 3 Evidence-Based Medicine Center, School of Basic Medical Sciences, Lanzhou University, Lanzhou, 730000 China; 4 Department of Health Research Methods, Evidence and Impact, McMaster University, Hamilton, ON Canada; 5 Guangdong Provincial Academy of Chinese Medical Sciences, Guangdong Provincial Hospital of Chinese Medicine, Guangzhou, 510006 China

**Keywords:** Depression, Therapeutics

## Abstract

Acupuncture is an important alternative therapy in treating major depressive disorder (MDD), but its efficacy and safety are still not well assessed. This study is the first network meta-analysis exploring the effectiveness and safety of acupuncture, common pharmacological treatments or other non-medication therapies for MDD. Eight databases including PubMed, Embase, Allied and Complementary Medicine Database, Cochrane Library, Wan Fang Data, China National Knowledge Infrastructure, China Biology Medicine disc, and Chongqing VIP Database were searched up to Jan 17, 2021. Articles were screened and selected by two reviewers independently. We used the Grading of Recommendations Assessment, Development and Evaluation (GRADE) approach to assess the certainty of the evidence. A total of 71 eligible studies were included. The network analysis results indicated that the combined interventions of electro-acupuncture (EA) with selective serotonin reuptake inhibitors (SSRIs) and manual acupuncture (MA) with SSRIs were more effective in improving depression symptoms compared with acupuncture alone, pharmacological interventions alone, or other inactive groups. Among all the regimens, EA with SSRIs was found to have the highest effect in improving depression symptoms of MDD. In addition, there were slight differences in the estimations of the various treatment durations. The combination of acupuncture and serotonin-norepinephrine reuptake inhibitors (SNRIs) was found to be more effective than SNRIs alone. In conclusion, acupuncture and its combinations could be safe and effective interventions for MDD patients. EA with SSRIs seems to be the most effective intervention among the assessed interventions. Well-designed and large-scale studies with long-term follow-up should be conducted in the future.

## Introduction

Major Depressive Disorder (MDD) is a serious mood disorder characterized as depressive mood and loss of interest. MDD affects up to 3.0% (2.4–3.8%) of the population worldwide^1^. In the United States (US), the 12-month prevalence of MDD is approximately 7%, and the rate in females could even be 1.5–3 folds higher than males at the early time of adolescence^2^. Diagnosis of MDD requires a period of major depressive episode which shows depressed mood, and loss of interest nearly every day for at least 2 weeks^3^. With the high recurrence of MDD (35 and 85% in the general population and specialized mental health care settings respectively after 15 years)^4^, uncontrolled and severe MDD causes continuously suicidal behaviors and creates extra medical and economic burdens^5,6^.

The second-generation antidepressants (SGAs), selective serotonin reuptake inhibitors (SSRIs), serotonin-norepinephrine reuptake inhibitors (SNRIs), etc., are considered and commonly applied as first-line treatment options for MDD^7^. However, side effects and non-response can occur commonly^8^. Patients treated with SNRIs complained of side effects such as sleep disturbances, sexual dysfunction, appetite changes, and headache^9^. Dizziness, fatigue, constipation, and dry mouth occur more frequently in patients using tricyclic antidepressants (TCAs)^10^. 30–50% of the patients show non-response to the treatment with antidepressants^11^. Due to these reasons, a variety of nonpharmacological approaches, including psychology consulting and complementary and alternative medicine (CAM), are adopted for the treatment of MDD. And acupuncture is one of the most commonly used nonpharmacological treatments. In the US, it is estimated that 0.6% of patients suffering from severe depression choose acupuncture^12^.

In recent decades, existing systematic reviews and meta-analyses suggested combination of acupuncture and SSRIs or SNRIs in treating MDD patients. As network meta-analysis (NMA) is a more efficient approach in evaluating and ranking multiple interventions, we conducted this study to assess the effectiveness and safety of different techniques of acupuncture in treating patients with MDD.

## Methods

### Search strategy for identification of studies

The systematic search was conducted in eight databases, PubMed, Embase, Allied and Complementary Medicine Database (AMED), Cochrane Library, Wan Fang Data, China National Knowledge Infrastructure (CNKI), China Biology Medicine disc (CBM, CBMdisc), and Chongqing VIP Database (CQVIP), from their inception to Jan 17, 2021. The following terms were used in the search strategies: (Acupuncture, Acupuncture Therapy, Electroacupuncture, Acupuncture, needling, electrostimulation, auriculoacupuncture, Electro-acupuncture, Electroacupuncture) and (depression, depressive disorder). The search strategies were adapted and specified for different databases. Details of the search strategies were listed in the Supplementary Method.

Preferred Reporting Items for Systematic Reviews and Meta-Analyses (PRISMA) and its extension statement, the PRISMA Extension Statement for Reporting of Systematic Reviews Incorporating Network Meta-analyses of Health Care Interventions (PRISMA-NMA), were regarded as the templates when reporting this systematic review and network meta-analyses^13,14^. This study was registered in PROSPERO, number CRD42019136229.

### Study selection

Two reviewers (Z.C. Hu and L. Yao) independently evaluated studies for inclusion. Any disagreements were reviewed by the third reviewer (L.L.D. Zhong) and resolved by discussion among all reviewers. Studies that met the following criteria were included: (1) randomized control trials (RCTs) that adopted a double-blind, single-blind, or quasi-blind design; (2) patients met established diagnostic criteria of major depressive disorder, including the Diagnostic and Statistical Manual of Mental Disorders (DSM), the International Classification of Diseases (ICD) and the Chinese Classification of Mental Disorders (CCMD); (3) types of acupuncture were included: manual acupuncture (MA), electro-acupuncture (EA); (4) acupuncture alone or combined with antidepressant medications was compared with antidepressant medications, blank control, waitlist control, placebo control, or other non-medication therapies. Studies with the diagnosis of post-stroke depression, postpartum depression, depression during pregnancy, and depression due to the general medical condition were excluded.

### Data abstraction

Two independent reviewers (Z.C. Hu and W.Y. Huang) extracted data from selected RCTs. Characteristics such as first author, titles of study, participants (gender, age, duration, sample sizes), study design (randomization, blinding), interventions, control interventions, outcome measures, results, and adverse events were recorded in a pre-made form. Pharmacological treatments evaluated were sorted by the five main antidepressants types: SSRIs, SNRIs, TCAs, monoamine oxidase inhibitors (MAOIs), noradrenaline and specific serotoninergic antidepressants (NASSAs). Acupuncture treatments were sorted by EA, MA, sham EA, and sham MA. Any disagreements were reviewed by the third reviewer (W.C. Lam) and resolved by discussion among all reviewers.

### Outcomes

Hamilton Depression Rating Scale (HDRS, also abbreviated as HAMD) and Self-Rated Depression Scale (SDS) were defined as the primary efficiency outcome measures. Side Effect Rating Scale (SERS), Treatment Emergent Symptom Scale (TESS), and the number of adverse events or patients dropping out of the study due to any reason were defined as the primary safety outcome. Other assessment questionnaires measuring the depression level of MDD patients were collected at the same time.

### Quality assessment

The identified trials were assessed independently by two reviewers (W.C. Lam and L. Yao). The risks of bias of the included RCTs were assessed using Revised Cochrane risk-of-bias tool for randomized trials (RoB 2)^15^. The appraisal of acupuncture procedure was based on the criteria of the Revised Standards for Reporting Interventions in Clinical Trials of Acupuncture (STRICTA)^16^. Any disagreements were reviewed by the third reviewer (L.L.D. Zhong) and resolved by discussion among all reviewers.

### Data synthesis and analysis

A network plot was constructed to illustrate all the relationships of the included interventions. Nodes represented the competing treatments, and edges represented the available direct comparisons between pairs of treatments. The size of the node and the width of the edges in the network plot were both weighted according to the number of studies involved in each direct comparison. The effects of multiple interventions were compared by estimating mean differences (MDs) on the change score between final and baseline scores on depression symptoms measured by the same scales. For studies that did not report the mean change from baseline, we calculated the mean change score in each intervention arm as the mean final score minus mean baseline score. For a trial that did not report the standard deviation (SD) of the change score, it was computed as $\sqrt{{SD}_{B}^{2}}+{SD}_{F}^{2}+2 \times r \times {SD}_{B} \times {SD}_{F}$, where SDB and SDF were the SDs of the baseline and final scores, and a moderate correlation coefficient of r = 0.5 between baseline and final irritability score was assumed. Since a higher score represents worse depression symptoms and the change score was defined as the final minus baseline score. A treatment was considered more efficacious than another treatment if the corresponding estimate of MD on the change score was negative and the 95% confidence interval (CI) did not include zero. The NMA was conducted based on the same scale to decrease potential heterogeneity and ensure the similarity of the outcomes data.

Bayesian NMAs with the package ‘gemtc’ V.0.8.1 of RStudio software (ver. 0.96.315; RStudio Inc, Boston, MA, USA) was performed to compare the effects of different prophylactic agents. The Markov Chains Monte Carlo sampler was used to generate samples. A total of 10 000 simulations for each chain was set as the ‘burn-in’ period. Posterior summaries were based on 100 000 subsequent simulations. Model convergence was assessed using the Brooks–Gelman–Rubin plots method. Global heterogeneity was assessed on the bias of the magnitude of heterogeneity variance parameter estimated from the NMA models using the mtc.anohe command of the ‘gemtc’ package. The normal likelihood used for the mean change score was continuous^17^. A random-effects network meta-analyses were performed for the NMA to account for the potential heterogeneity in the data. The comparative efficacies between the antimanic drugs were expressed using sham MA as reference.

A node splitting method was used to examine the inconsistency between direct and indirect comparisons when a loop connecting three arms exists^18^. The ranking probabilities for all treatments were also estimated, and a treatment hierarchy using the probability of being the best treatment was obtained^19^. This process was performed using the cumulative ranking curve (SUCRA). The SUCRA index ranged between 0 and 1, where the treatments with higher SUCRA values were considered to have better efficacy. Moreover, the subgroup analyses were conducted according to the different treatment duration to further explore the potential resource of heterogeneity. All outcomes from included studies were divided into three groups based on the duration of treatment, short-term as 1 ≤ x ≤ 4 weeks, mid-term as 4 < x ≤ 8 weeks, and long-term as x > 8 weeks.

### Assessing certainty of the evidence

The Grading of Recommendations Assessment, Development and Evaluation (GRADE) were used to assess the certainty of the direct, indirect, and network estimates for all outcomes. The certainty of direct evidence of the randomized trials starts from high and can be rated down to be moderate, low and very low^20^. Certainty ratings of indirect estimates start at the lowest GRADE rating of the direct comparisons that contributed to the most-dominant first order loop, with a further rating down for intransitivity when present^21,22^. Ratings of the certainty of estimates for direct and indirect estimates to inform the rating of network estimates include risk of bias, inconsistency, indirectness, and publication bias, while imprecision was assessed at the network level. For the certainty of network estimates, we started with the estimate—direct or indirect—that dominates (contribution > 50%) the network estimate or use the higher of the direct and indirect estimates if they both contributed importantly to the network estimate. If incoherence is present, when both the direct and indirect evidence has the same certainty of evidence: we used the network estimate, but rate down the certainty of evidence; when the direct and indirect evidence does not have the same certainty of evidence: we used the higher certainty evidence instead of the network estimate. We used the MAGICapp platform to develop GRADE summary of finding tables for each outcome.

## Results

### Study identification

**Figure 1:**
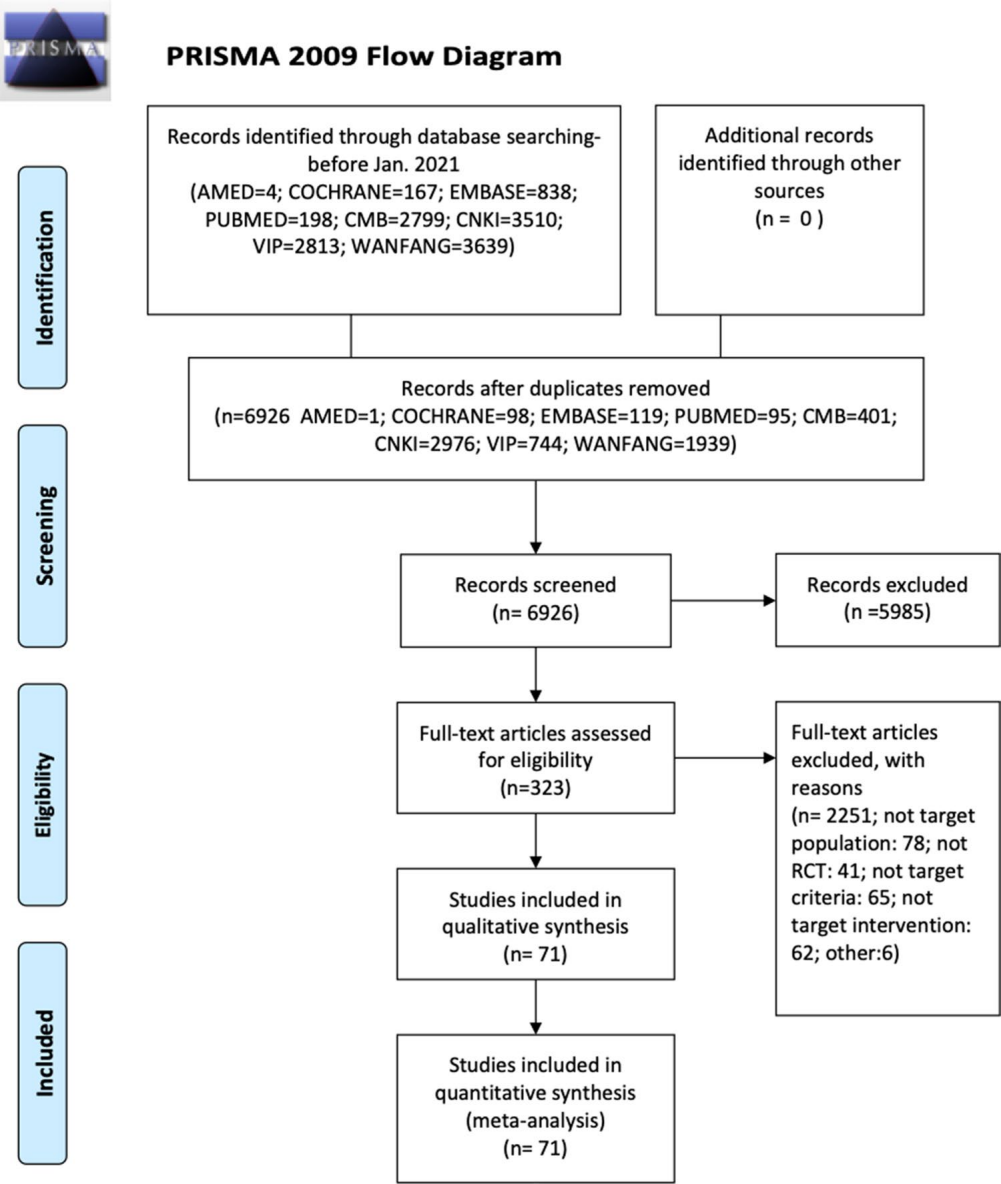
Flowchart of literature selection on systematic reviews on acupuncture for treating major depressive disorder.

The flow diagram of literature selection was shown in Fig. 1 with reasons for exclusion at each stage. According to the prespecified selection criteria, 71 eligible studies and a total of 5856 individuals were assessed with eligibility and included in the review.

### Characteristics of the included studies

The aggregated characteristics of the included RCTs were shown in Table 1. 16 studies^3,24,31,34,37,49,53,56,60,65,75,76,83,86–88^ met DSM (III revision: 1; IIIR revision: 1; IV revision: 10; V revision: 4), 45 studies^26–29,32,33,35,36,38–42,44–46,48,50,53,54,57–59,61–63,66–70,74,77–82,84,85,88–90,92,93^ met CCMD (3rd version: 44; 2R: 1), 14 studies^25,30,40,43,47,51,52,55,64,71–73,80,91^ met ICD (10th revision: 13; 9th revision: 1).

**Table 1:** The aggregated characteristics of the included RCTs.

Source		Study design	Population			Treatment						Outcome measures
		No. of arm	Diagnosis criteria	No	Age	Intervention Group (type; duration; frequency; or drug name; dose; duration; frequency)		Comparator Group (type; duration; frequency; or drug name; dose; duration; frequency)		Third Group (type; duration; frequency; or drug name; dose; duration; frequency)		
Ai et al.^23^	2018	2	DSM-V	I: 50; C: 50	I: 20.1 ± 3.6; C: 20.2 ± 3.5	MA + paroxetine	MA: 1 time/ d, 6 w; paroxetine: 20 mg/time, 1 time/ d, 6 w	Paroxetine	20 mg/time, 1 time/ d, 6 w	N/A	N/A	HAMD-17(0, 1, 2, 4, 6 w); response rates
Allen et al.^24^	2006	3	DSM-IV	I: 53; C: 52; T: 52	I: 23.3 ± 11.4; C:24.6 ± 12.8; T: 22.7 ± 14.0	MA	2 times/ w for first 4 w, 1 time/ w for another 4 w, 8 w	MA	2 times/ w for first 4 w, 1 time/ w for another 4 w, 8 w	Wait-list	8w	HAMD-17 (0, 4, 8, 12, 16 w), BDI (0, 1, 2, 3, 4, 5, 6, 7, 8, 9, 10, 11, 12, 13, 14, 15, 16 w); response rates (8, 16 w), remission rates (8, 16 w)
Chen et al.^25^	2014	2	ICD-10	I: 40; C: 33	I: 36.2 ± 11.7; C: 35.0 ± 10.5	MA + Paroxetine	MA: 3 times/ w, 6 w; Paroxetine: 1 time/ d, 10–20 mg/ time, 6 w	Paroxetine	1 time/ d, 10–20 mg/ time, 6 w	N/A	N/A	HAMD-17(0, 1, 2, 4, 6w), SERS( 2, 4, 6w); response rates
Chen et al.^26^	2010	2	CCMD-III	I: 33; C: 30	I:65.30 ± 3.592; C: 65.10 ± 3.736	MA	5 time/ w, 6 w	Fluoxetine	1 time/ d, 20 mg/ time, 6 w	N/A	N/A	HAMD(0, 2, 4, 6w); response rates
Chen et al.^27^	2011	2	CCMD-III	I: 30; C: 30	I: 43.9 ± 11.2; C: 49.0 ± 13.4	EA + Fluoxetine	EA: 6 times/ w, 8 w; Fluoxetine: 1 time/ d, 20 mg/ time, 8 w	Fluoxetine	1 time/ d, 20 mg/ time, 8 w	N/A	N/A	HAMD-17(0, 1, 4, 8 w); response rates
Dong et al.^28^	2017	2	CCMD-III	I: 30; C: 30	I: 15.6 ± 1.40; C: 14.9 ± 1.45	MA + Psychotherapy	MA: 1 time/ d, 10 d one session, another session after 2d, 30 d; Psychotherapy: 1 times/ w, 4 w	Sertraline + Psychotherapy	Sertraline: 1 time/ d, 50 mg/ time, 30 d; Psychotherapy: : 1 times/ w, 4 w	N/A	N/A	HAMD-24(0, 10, 20, 30d)
Duan et al.^29^	2008	3	CCMD-III	I: 25; C: 25; T: 25	I: 50.12 ± 4.32; C: 49.72 ± 5.47; T: 48.93 ± 7.60	EA	6 time/ w, 6 w	Fluoxetine	20 mg/ d, 6 w	EA + Fluoxetine	EA: 6 time w, 6 w; Fluoxetine: 20 mg/ d, 6 w	HAMD(0, 6w), TESS (2, 4, 6w); response rates
Feng et al.^30^	2015	3	ICD-10	I: 60; C: 60; T: 60	I: 36.4 ± 9.9; C: 37.1 ± 9.8; T: 36.6 ± 10.0	MA + SSRIs	MA: 3 times/ w, 6 w; Fluoxetine: 20–60 mg/ d; or Paroxetine: 20–60 mg/ d; or Citalopram: 20–60 mg/ d; or Sertraline: 50–200 mg/ d; or Fluvoxamine: 50–300 mg/d; 1–2 times/ d, 6w	SSRIs	Fluoxetine: 20–60 mg/ d; or Paroxetine: 20–60 mg/ d; or Citalopram: 20–60 mg/ d; or Sertraline: : 50–200 mg/ d; or Fluvoxamine: 50–300 mg/d; 1–2 times/ d, 6w	HC	HC	MADRS(0, 1, 2, 4, 6w); SERS(1, 2, 4, 6w)
Gallagher et al.^31^	2001	3	DSM-IV	38	18–45	MA	8 w	MA	8 w	Wait-list	8 w	HRSD-19(0, 8 w)
Gu et al.^32^	2015	2	CCMD-III	I: 30; C: 30	I: 61.83 ± 10.33; C: 63.53 ± 10.11	MA	1 time/ d, 30 d	Fluoxetine	1 time/ d, 20 mg/ time, 30 d	N/A	N/A	HAMD-17(0, 30d)
Guo et al^33^	2019	2	CCMD-3	I: 22; C: 22	I: 40.86 ± 9.84; C: 42.09 ± 10.71 years	MA + Fluoxetine	MA: 1 time/ d, 3w; Fluoxetine: 20 mg/ time,1 time/ d, 3 W	Fluoxetine	20 mg/ time,1 time/ d, 3 W	N/A	N/A	HAMD-17, PHQ-9(0, 3w); response rate
Han et al^34^	2019	2	DSM-V	I: 25; C: 25	I: 37.0 (32. 0, 41. 5); C: 39.0 (35.0, 46.5)	EA	3 times/ w, 6 w	MA	3 times/ w, 6 w	N/A	N/A	HAMD, SDS(0,6w); response rate
Huang et al.^35^	2013	2	CCMD-III	I: 30; C: 30	I: 49.25 ± 14.03; C: 50.78 ± 12.96	MA + Paroxetine	MA: 3 times/ w, 6 w; Paroxetine: 1 time/ d, 20–40 mg/ time, 6 w	Paroxetine	1 time/ d, 20–40 mg/ time, 6 w	N/A	N/A	HAMD(0, 1, 6w), SDS(0, 1, 6w); Asberg(1, 6w); response rates
Jiang et al.^36^	2008	2	CCMD-III	I: 34; C: 34	I: 36.4 ± 11.7; C: 35.6 ± 13.1	MA + Citalopram	MA: 1 time/ 2 d, 40 d Citalopram: 20 mg/ d, 1 time/ d, 40 d	Citalopram	20 mg/ d, 1 time/ d, 40 d	N/A	N/A	HAMD-17(0, 1, 2, 4, 6w), CGI-SI(0, 2, 4, 6w); TESS; response rates
Li et al.^37^	2013	2	DSM-IV	I: 62; C: 62	I: 33.9 ± 12.0; C: 34.8 ± 12.4	EA + Sertraline	EA: 6 times/ w, 8 w; Sertraline: 50–100 mg/ d, 8 w	Sertraline	50–100 mg/d, 8 w	N/A	N/A	HAMD-17(0, 1, 2, 4, 8w)
Li et al.^38^	2004	3	CCMD-III	I: 30; C: 30; T: 50	I: 41.8 ± 14.6; C: 39.4 ± 13.4; T: 45.8 ± 14.5	MA	5 times/ w, 6 w	Fluoxetine	1 time/ d, 20 mg/ time, 6 w	MA 2	5 times/ w, 6 w	HRSD, SDS(0, 6 w); response rates
Lin et al.^39^	2004	2	CCMD-III	I: 29; C: 28	I: 40.3 ± 11.5; C: 44.6 ± 12.7	MA + Paroxetine	MA: 1 time/ d, 5 time/ w, 6 w; Paroxetine: 1 time/ d, 10 mg/ time, 6 w	Paroxetine	1 time/ d, 20 mg/ time, 6 w	N/A	N/A	HAMD-17(0, 1, 2, 4, 6w), HAMA(0, 1, 2, 4, 6w); CGI TESS; response rates
Li et al.^40^	2017	2	ICD-10, CCMD-III	I: 30; C: 30	I: 34 ± 8; C:35 ± 8	MA + Paroxetine	MA: 6 time/ w, 8 w; Paroxetine: 10- 20 mg/ time, 1 time/ d, 8w	Paroxetine	10- 20 mg/ time, 1 time/ d, 8w	N/A	N/A	HAMD-17(0,1,2,3,4,5,6,7,8w); response rates
Lin et al.^41^	2005	2	CCMD-III	I: 30; C: 23	I: 41.7 ± 12.1; C: 43.1 ± 11.5	MA + Fluoxetine	MA: 5 time/ w, 6 w; Fluoxetine: 1 time/ d, 20 mg/ time, 6 w	Fluoxetine	1 time/ d, 20 mg/ time, 6 w	N/A	N/A	HAMD-17(0, 1, 2, 4, 6w), HAMA(0, 1, 2, 4, 6w); CGI, TESS; response rates
Lin et al.^42^	2014	2	CCMD-III	I: 61; C: 61	NR	MA + Fluoxetine	1 time/ d, 5 time/ w, 4 w	Fluoxetine	1 time/ d, 20 mg/ time, 4 w	N/A	N/A	HAMD-17(0, 1, 2, 4w), SDS(0, 4w ); response rates
Liu et al.^43^	2018	2	ICD-10	I: 21; C: 21	I: 44 ± 10; C:43 ± 9	Fluoxetine + MA	MA: 1 time/ d for first 3d, then once every 3d, 8 w; Fluoxetine: 10 mg, 1 time/ d, 8w	Fluoxetine + Sham MA	Sham MA: 1 time/ d for first 3d, then once every 3d, 8 w; Fluoxetine: 10 mg, 1 time/ d, 8w	N/A	N/A	MADRS(0, 4, 8w), SDS(0, 4, 8w); response rates
Liu et al.^44^	2005	2	CCMD-III	I: 21; C: 20	I: 48.9 ± 12.0; C: 49.0 ± 13.4	EA + SSRIs*	EA: 6 w; SSRIs: 6 w*	SSRIs*	SSRIs: 6 w*	N/A	N/A	HAMD-17(0, 1, 2, 4, 6w)
Liu et al.^45^	2014	3	CCMD-III	I: 45; C: 45; T: 45	I: 47.11 ± 8.32; C:47.58 ± 8.21; T: 48.11 ± 7.97	MA + SSRIs	MA: 1 time/ 2 d, 4 w; Fluoxetine: 20 ~ 60 mg/ d; or Paroxetine: 20–60 mg/ d; or Citalopram: 20–10 mg/ d; or Sertraline: 50–200 mg/ d; 1time/ d, 4 w	SSRIs	Fluoxetine: 20 ~ 60 mg/ d; or Paroxetine: 20–60 mg/ d; or Citalopram: 20–10 mg/ d; or Sertraline: 50–200 mg/ d; 1time/ d, 4 w	HC	HC	HAMD-17(0, 1, 2, 4w), SERS(1, 2, 4w); response rates
Liu et al.^46^	2015	2	CCMD-III	I: 45; C: 45; T: 45	I: 36 ± 11; C: 37 ± 11; T: 36 ± 11	MA + SSRIs	MA: 1 time/ 2 d, 4 w; SSRI: Fluoxetine: 20–60 mg/ d; or Paroxetine: 20–60 mg/ d; or Citalopram: 20–60 mg/ d; or Sertraline: 50–200 mg/ d; or Fluvoxamine: 50–300 mg/d; 1–2 times/ d, 4 w	SSRIs	Fluoxetine: 20 ~ 60 mg/ d; or Paroxetine: 20–60 mg/ d; or Citalopram: 20–60 mg/ d; or Sertraline: 50–200 mg/ d; or Fluvoxamine: 50–300 mg/d; 1-2times/ d, 4 w	HC	HC	HAMD-17(0, 1, 2, 4w)
Liu et al.^47^	2017	3	ICD-10	I: 47; C: 48; T: 45	I: 47.11 ± 9.10; C:47.27 ± 9.03; T:47.18 ± 9.21	MA + Venlafaxine	MA: 5 times/ w, 8w; Venlafaxine: 75–225 mg/ d, 8w	Venlafaxine	75–225 mg/ d, 8w	HC	HC	HAMD-17(0, 8w)
Lu et al.^48^	2017	2	CCMD-III	I: 30; C: 30	I:52.7 ± 17.4; C:56.7 ± 16.3	MA + Escitalopram	EA: 3 times/ w, 2 w; Escitalopram :20 mg, 1 time/ d, 2w	Escitalopram	20 mg, 1 time/ d, 2w	N/A	N/A	HAMD(0, 1, 2w),
Luo et al.^49^	2003	3	DSM-IV	I: 31; C: 32; T: 32	I: 30 ± 11; C: 34 ± 13; T: 32 ± 12	EA + Placebo	EA: 1 time/ d, 5 time/ w; Placebo: 1 time/ d, 20 mg/ time	Fluoxetine + Sham EA	Fluoxetine: 1 time/ d, 20 mg/ time; Sham EA: 1 time/ d, 5 time/w	Placebo + sham-EA	Placebo: 1 time/ d, 20 mg/ time; sham-EA: 1 time/ d, 5 time/ w	HAMD, SDS, CGI(0, 2, 4, 6w); Asberg(0, 6w)
Ma et al.^50^	2011	2	CCMD-III	I: 31; C: 29	I: 51.1 ± 12.85; C:50.9 ± 11.29	MA	5 time/ w, 6 w	Fluoxetine	1 time/ d, 20 mg/ time, 6 w	N/A	N/A	HAMD-17(0, 2, 4, 6w), Asberg(2, 4, 6w); response rates
Ma et al.^51^	2011	2	ICD-10	I: 26; C: 29	I: 46.27 ± 13.13 C: 40.52 ± 14.21	EA + Paroxetine	EA: 3 times/ w, 6w; Paroxetine: 10–20 mg/ d, 1times/ d, 6w	Paroxetine	10–20 mg/ d, 1times/ d, 6w	N/A	N/A	HAMD-17, SERS(0, 1, 2, 4, 6w), CGI(0, 6 w)
Ma et al.^52^	2011	2	ICD-10	I: 26; C: 29	I: 46.27 ± 13.13 C: 40.52 ± 14.21	EA + Paroxetine	EA: 3 times/ w, 6 w; Paroxetine: 10–20 mg/ d, 1 time/ d, 6w	Paroxetine	10–20 mg/ d, 1 time/ d, 6w	N/A	N/A	HAMD-17, SDS(0, 1, 2, 4, 6w)
Ma et al^53^	2020	2	CCMD-3/DSM-V	I: 30; C: 32	22–70	MA	3 times/ w, 8w	Sham MA	3 times/ w, 8w	N/A	N/A	HAMD, SDS, TESS(0, 4, 8,12w)
Pei et al.^54^	2006	2	CCMD-III	I: 62; C: 58	I: 18–61; C:20–64	MA	5 time/ w, 6 w	Fluoxetine	1 time/ d, 20 mg/ time, 6 w	N/A	N/A	HAMD(0, 2, 4, 6w); response rates
Qu et al.^55^	2013	3	ICD-10	I: 58; C: 54; T: 48	I: 33.2 ± 9.0; C: 32.3 ± 9.6; T: 34.4 ± 10.8	EA + Paroxetine	EA: 3 times/ w, 6 w; Paroxetine:10–20 mg/ d, 6 w	MA + Paroxetine	MA: 3 times/ w, 6 w; Paroxetine:10–20 mg/ d, 6 w	Paroxetine	Paroxetine:10–20 mg/ d, 6 w	HAMD-17 (0, 1, 2, 4, 6w, 10w follow-up), SDS (0, 1, 2, 4, 6w, 10w follow-up), CGI-S (0, 1, 2, 4, 6w, 10w follow-up); response rates
Roschke et al.^56^	2000	3	DSM-III-R	I: 22; C: 24; T: 24	I: 49 ± 13; C: 47 ± 9; T: 49 ± 11	MA + Mianserin	MA: 3 times/ w, 4 w; Mianserin: 90–120 mg/ d, 4 w ;	Sham MA + Mianserin	Mianserin: 90–120 mg/ d, 4 w; Sham MA: 3 times/ w, 4 w	Mianserin	90–120 mg/ d, 4 w	GAS, BRMS, CGI, Bf-S (twice/ w for 8 w); response rates
Shi et al.^57^	2015	3	CCMD-III	I: 30; C: 30; T: 30	I: 52.33 ± 9.93; C: 48.46 ± 8.44; T: 49.94 ± 9.41	MA	5 times/ w, 8 w	MA	5 times/ w, 8 w	Fluoxetine	1 time/ d, 20 mg/ time, 8 w	HAMD-17(0, 4, 8w); response rates
Song et al.^58^	2013	2	CCMD-III	I: 30; C: 30	I: 42.32 ± 12.47; C: 43.74 ± 12.52	MA	6 times/ w, 6 w	Fluoxetine	1 time/ d, 20 mg/ time, 6 w	N/A	N/A	HAMD(0, 2, 4, 6w), Asberg (2, 4, 6w); response rates
Sun et al.^59^	2012	2	CCMD-III	I: 20; C: 20	I: 32.5 ± 10.3; C: 31.5 ± 11.4	EA + Venlafaxine	EA: 5 times/ w, 2 w; Venlafaxine: 1 time/ d, 75–150 mg/ time, 2 w	Venlafaxine	1 time/ d, 75–150 mg/ time, 2 w	N/A	N/A	HAMD(0, 1, 2w); TESS; response rates
Sun et al.^60^	2013	3	DSM-IV	I: 25; C: 25; T: 25	I: 43.10 ± 13.86; C:42.56 ± 10.70; T:40.72 ± 12.80	EA	5 times/ w, 6 w	EA	5 times/ w, 6 w	Fluoxetine	20 mg/ d, 6 w	HDRS-24 (0, 2, 4, 6 w)
Tang et al.^61^	2003	2	CCMD-IIR	I: 32; C: 32	I: 18–51; C: 19–56	EA + Amitriptyline	EA: 1w-2w: 7 times/ w; 3w-6w: 3 times/ w; 6w; Amitriptyline: 1 time/ d, 50 mg/ time, 6 w	Amitriptyline	2 times/ d, 25–75 mg/ time, 6 w	N/A	N/A	SDS(0, 3, 6w), SAS(0, 3, 6w); response rates
Tian et al.^62^	2008	2	CCMD-III	I: 30; C: 30	I: 35.1 ± 14.3; C: 34.8 ± 15.1	EA + Clomipramine*	EA: 5 times/ w, 6 w; Clomipramine: 1 time/d , 25–75 mg/ time, 6 w*	Clomipramine*	1 time/d , 25–250 mg/ time, 6w;	N/A	N/A	HAMD-17(0, 6 w); response rates
Wang et al.^63^	2018	2	CCMD-III	I: 40; C: 40	I: 34.2 ± 10.9; C: 33.4 ± 11.8	MA + Fluvoxamine	5 times/ w, 6w; Fluvoxamine: 100 ~ 150 mg/ d, 6 w	Fluvoxamine	100 ~ 150 mg/ d, 6 w	N/A	N/A	HAMD-24(0, 2, 4, 6w), SERS(1, 2, 4, 6w); response rates
Wang et al.^64^	2014	3	ICD-10	I: 23; C: 32; T:17	I: 47 ± 11; C: 45 ± 12; T: 48 ± 9	EA + Paroxetine	EA: 1 time/ 2 d, 6 w; Paroxetine: 10–20 mg/ d, 1 time/ d, 6 w	MA + Paroxetine	MA: 3 times/w, 6 w; Paroxetine: 10–20 mg/ d, 1 time/ d, 6 w	Paroxetine	10–20 mg/ d, 1 time/ d, 6 w	HAMD-17(0, 1, 2, 4, 6w), SERS(0, 2, 4, 6w), WHOQOL—BREF(0, 6w); response rates
Wang et al.^65^	2016	2	DSM-IV	I: 32; C: 32	I:41.3 ± 5.2;C:42.1 ± 4.7	MA	1 time/ d, 6 time/ w, 8 w	Mirtazapine	1 time/ d, 15–45 mg/ time, 8 w	N/A	N/A	HAMD-24(0, 4, 8w); Asberg; response rates
Wang et al.^66^	2007	3	CCMD-III	I: 35; C: 35	I: 17–65; C: 19–63	EA	5 times/ w, 12 w	Sertraline	25–75 mg/ d, 12 w	HC	HC	HAMD-17(0, 2, 4, 8, 12)
Wang et al.^67^	2007	2	CCMD-III	I: 30; C: 30	I: 18–68; C: 17–70	EA*	5 times/ w, 12 w*	Sertraline*	25–7 5 mg/ d, 1 time/ d, 12 w*	N/A	N/A	HAMD-17(0, 2, 4, 8, 12)
Wang et al.^68^	2006	2	CCMD-III	I: 38; C: 38	I: 18–65; C: 19–63	EA*	5 times/ w, 24 w*	Sertraline*	25–75 mg/d, 1 time/ d, 24 w*	N/A	N/A	HAMD-17(0, 6, 12, 24); response rates
Wang et al.^69^	2007	2	CCMD-III	I: 50; C: 50	I: 17–80; C: 19–73	EA*	5 time/ w, 12 w*	Sertraline*	1time/ d, 25–75 mg/ time, 12 w*	N/A	N/A	HAMD-17(0, 2, 4, 8, 12w); response rates
Wang et al.^70^	2008	2	CCMD-III	I: 50; C: 42	I: 52.8 ± 14.1; C: 52.1 ± 15.4	MA + SSRIs*	MA: 5 time/w, 4w; SSRIs*	SSRIs*	SSRIs*	N/A	N/A	HAMD-17, SDS(0, 1, 2, 4w ); response rates
Wang et al.^71^	2010	2	ICD-10	I: 30; C: 30	I: 35.7 ± 11.1 C: 41.2 ± 9.0	EA + Fluoxetine	EA: 7 times/ w, 6 w; Fluoxetine: 20 mg/ d, 1 time/ d, 6 w	Fluoxetine	20 mg/ d, 1 time/ d, 6 w	N/A	N/A	HAMD-17(0, 2, 4, 6w); response rates
Wang et al.^72^	2014	2	ICD-9	I: 47; C: 29	NR	MA + SSRIs/ SNRIs	MA: 5 times/ w, 6 w; Fluoxetine: 20 mg, 1 time/ d; or Paroxetine: 20 mg, 1 time/d; r Duloxetine: 40 mg, 1 time/ d, 6w	SSRIs/ SNRIs	Fluoxetine: 20 mg, 1 time/ d; or Paroxetine: 20 mg, 1 time/d; r Duloxetine: 40 mg, 1 time/ d, 6w	N/A	N/A	HAMD-17 (0, 1, 2, 4, 6 w)
Wang et al.^73^	2017	2	ICD-10	I: 22; C: 24	I: 44.5 ± 10.47; C: 43.78 ± 9.10	MA + Fluoxetine	MA : 1 time/d for the first three days, 1 time/ 3 d for the reminder of the 8-w trial; Fluoxetine: 20 mg/ d	Sham MA + Fluoxetine	Sham MA : 1 time/d for the first three days, 1 time/ 3 d for the reminder of the 8-w trial; Fluoxetine: 20 mg/ d	N/A	N/A	MADRS, SDS(0, 8 w)
Wang et al^74^	2020	2	CCMD-3	I: 48; C: 48	I: 34.19 ± 8.4; C: 32.71 ± 8.2	Venlafaxine + MA*	MA: 3 times/ w, 12 w; Venlafaxine: 1st w:75 mg/d, 2ed w:150 mg/d, 3-6th w 225 mg/d, 12w*	Venlafaxine*	MA: 3 times/ w, 12 w; Venlafaxine: 1st w:75 mg/d, 2ed w:150 mg/d, 3-6th w 225 mg/d, 12w*	N/A	N/A	HAMD-17, SERS(0, 4, 8, 12 w); response rates
Wang et al^75^	2019	3	DSM-V	I: 30; C: 30; T:30	I: 32 ± 8; C: 32 ± 7;32 ± 8	Venlafaxine + MA*	MA: 3 times/ w, 12 w; Venlafaxine:1st w:75 mg/d, 2ed w:150 mg/d, 3-6th w 225 mg/d, 12w*	Venlafaxine *	1st w:75 mg/d, 2ed w:150 mg/d, 3-6th w 225 mg/d, 12w*	HC	HC	HAMD-17, BDI(0,2,8,12w), SERS(2,8,12w)
Wang et al.^76^	2013	2	DSM-IV	I: 30; C: 30	I: 48.1 ± 13.40; C: 47.10 ± 10.60	EA	3 times/ w, 24 w	Paroxetine	20–60 mg/ d, 1 time/ d, 24 w	N/A	N/A	MMPI, MADRS, SDS, SAS(0, 24w)
Wen et al.^77^	2003	2	CCMD-III	I: 31; C: 30	I: 31.6 ± 13.6; C:32.7 ± 14.1	EA + SSRIs	EA: 1 time/ d, 6 w; SSRI	SSRIs	SSRIs	N/A	N/A	HAMD(0, 2, 4, 6w); response rates
Wu et al.^78^	2010	2	CCMD-III	I: 33; C: 33	I: 68.52 ± 4.84; C: 69.64 ± 5.19	EA + Citalopram	EA: 5 times/ w, 6w; Citalopram: 20–40 mg/ d, 6 w	Citalopram	20–40 mg/ d, 6w	N/A	N/A	HAMD-17, TESS (0, 1, 2, 4, 6 w); response rates
Xu et al.^79^	2009	2	CCMD-III	I: 21; C: 20	I: 34; C: 31	MA	6 w	Fluoxetine	20 mg/d, 6 w	N/A	N/A	HAMD-17(0, 6 w); response rates
Xu et al.^80^	2004	2	CCMD-III、ICD-10	I: 30; C: 30	I: 42.5 ± 8.5; C:45.3 ± 9.2	MA	1 time/ d, 30d	Fluoxetine	20–40 mg/ d, 1 time/ d, 30d	N/A	N/A	HAMD-24(0, 1, 2, 4w)
Xu et al.^81^	2011	3	CCMD-III	I: 25; C: 30; T: 25	I: 47.51 ± 8.21; C: 47.42 ± 8.89; T: 48.01 ± 8.14	MA + SSRIs	MA: 3 times/ w, 6 w; Paroxetine, 20–60 mg, 1 time/ d, 6 w; or Sertraline: 50–200 mg, 1 time/ d, 6 w; or Fluoxetine: 20–80 mg, 1 time/ d, 6 w	SSRIs	Paroxetine,20–60 mg, 1 time/ d, 6 w; or Sertraline: 50–200 mg, 1 time/ d, 6 w; or Fluoxetine: 20–80 mg, 1 time/ d, 6 w	MA + Moxibustion + SSRIs	MA + Moxibustion: 3 times/ w, 6 w; Paroxetine,20–60 mg, 1 time/ d, 6 w; or Sertraline: 50–200 mg, 1 time/ d, 6 w; or Fluoxetine: 20–80 mg, 1 time/ d, 6 w	HAMD-17(0, 1, 2, 4, 6w); response rates
Yang et al.^82^	2012	3	CCMD-III	I: 30; C: 30; T: 30	I: 32.23 ± 13.98; C:33.40 ± 15.51; T:34.63 ± 12.71	EA + CBT	2 time/ w, 8 w; CBT: 1 time/w, 60-90 min/ time, 8 times	EA	2 time/ w, 8 w	CBT	1 time/w, 60-90 min/ time, 8 times	HAMD-17, CES-D(0.8w); response rates
Yi et al.^83^	2011	3	DSM-IV	I: 14; C:14; T:14	I: 37.0 ± 8.6; C:33.6 ± 8.4; T:35.5 ± 7.4	MA + Fluoxetine	MA: 5 time/w, 30 d; Fluoxetine: 1time/ d, 20 mg/ time, 30 d	Fluoxetine	1 time/ d, 20 mg/ time, 30 d	MA	5 time/ w, 30 d	HAMD-17(0, 30d)
Zhang et al.^84^	2007	2	CCMD-III	I: 38; C: 42	I: 41.57 ± 1.22; C: 39.82 ± 2.16	EA	1 time/ d, 6 w	Paroxetine	1 time/ d, 20 mg/ time, 6 w	N/A	N/A	HAMD-17(0, 2, 4, 6w)
Zhang et al.^85^	2012	2	CCMD-III	I: 20; C: 20	I: 47.58 ± 9.45; C: 45.65 ± 10.45	MA	4 w	Flupentixol + Melitracen	Flupentixol: 0.5 mg/time + Melitracen: 10 mg/time; 1 time/ d, 4 w	N/A	N/A	HAMD-17(0, 4w); response rates
Zhang et al.^86^	2007	2	DSM-III	I: 22; C: 20	I: 36.6 ± 9.7; C: 37.1 ± 10.2	EA + Paroxetine	EA: 6 times/ w, 6 w; Paroxetine: 10–40 mg/ d, 6 w	Paroxetine	10–40 mg/ d, 6 w	N/A	N/A	HAMD-17(0, 1, 2, 4, 6 w), TESS (1, 2, 4, 6 w); response rates
Zhang et al.^87^	2009	2	DSM-IV	I: 40; C: 40	I: 36.2 ± 11.7; C: 35.5 ± 12.0	MA + Fluoxetine (low dose) + placebo*	MA: 5 times/ w, 6 w; 10 mg fluoxetine + 1 placebo pill/ d in the first 2 w, followed by 10 mg fluoxetine + 2 placebo pills/d in the next 4 w*	Sham MA + Fluoxetine (normal dose)*	Sham MA: 5 times/ w, 6 w ; 20 mg/d in the first 2 w, followed by 30 mg fluoxetine/d in the next 4 w*	N/A	N/A	response rate, HRSD-17, HRSA (0, 2, 4, 6 w); SERS, acupuncture-specific side-effect checklist (2, 4, 6 w)
Zhao et al.^88^	2010	3	CCMD-III、DSM-IV	I: 30; C: 30; T: 30	I: 36.5 ± 14.7; C: 39.1 ± 12.3; T: 37.1 ± 12.3	EA	1 time/ d, 5 time/ w, 6w	EA	1 time/ d, 5 times/ d, 6 w	Fluoxetine	20 mg/ time, 1 time/ d, 6 w	HAMD-24(0, 2, 4, 6 w); response rates
Zhao et al.^89^	2010	2	CCMD-III	I: 48; C: 45	I: 40.9 ± 13.9; C: 41.5 ± 13.9	EA + Paroxetine*	EA: 7 times/ w, 3 w; Paroxetine: 10–40 mg/ d, 3 w*	Paroxetine*	10–40 mg/ d, 3 w*	N/A	N/A	HAMD(0, 3w); response rates
Zhao et al.^90^	2006	2	CCMD-III	I: 38; C:38	I: 18–65; C:19–63	EA*	5 time/ w, 12 w*	Sertraline*	1 time/ d, 25–75 mg/ time, 12 w*	N/A	N/A	HAMD-17(0, 1, 2, 8); response rates
Zhao et al.^91^	2019	3	ICD-10	I: 161; C: 160; T:156	I: 41.42 ± 12.53;C: 41.18 ± 12.00; T: 41.76 ± 12.85	MA + SSRIs	MA: 3 times/ w, 6 w; Paroxetine, Fluoxetine, Sertraline, Fluvoxamine, Citalopram, or Escitalopram: 10–20 mg/d, 6 w	EA + SSRIs	EA: 3 times/ w, 6 w; Paroxetine, Fluoxetine, Sertraline, Fluvoxamine, Citalopram, or Escitalopram :10–20 mg/d, 6 w	SSRIs	Paroxetine, Fluoxetine, Sertraline, Fluvoxamine, Citalopram, or Escitalopram: 10–20 mg/d, 6 w	response rate, remission rate(6w); early onset rate(1w), HAMD-17(0,1,2,4,6,10w), SDS(0,1,2,4,6,10w), CGI(6w), SERS(2,4,6w)
Zheng et al.^92^	2012	2	CCMD-III	I: 44; C: 54	I: 47.11 ± 9.52; C: 48.07 ± 10.09	MA	1 time/ 2d, 3 times/ w, 6 w	SSRI	Paroxetine,20–60 mg, 1 time/ d, 6 w; or Sertraline: 50–200 mg, 1 time/ d, 6 w; or Fluoxetine: 20–80 mg, 1 time/ d, 6 w	N/A	N/A	HAMD-17(0, 1, 2, 4, 6w, f), SERS(0, 1, 2, 4, 6w); response rates
Zhu et al.^93^	2018	2	CCMD-III	I: 33; C: 32	I: 42.9 ± 5.0; C:42.1 ± 4.3	MA + SSRIs*	MA: 5 time/ w, 6w; SSRIs, 6w*	SSRIs*	SSRIs, 6w*	N/A	N/A	HAMD-24,heart rate variability (0, 6w)

*NR* not reported, *N/A* not available, *I* intervention group, *C* comparator group, *T* third group, *HC* healthy central group, *d* day, *w* week, *SSRIs* selective serotonin reuptake inhibitors, *SERS/Asberg* total scores of rating scale for side effects, *PHQ-*9 patient health questionnaire-9, *HAMD/ HRSD/ HDRS* the Hamilton Depression Rating Scale, *CGI* the clinical global impression, *TESS* Treatment Emergent Symptom Scale, *MADRS* Montgomery–Asberg Depression Rating Scale, *SAS* Self-Rating Anxiety Scale, *SDS* Self-Rating Depression Scale, *MMPI* Minnesota Multiphasic Personality Inventory, *CES-D *The Center for Epidemiologic Studies Depression Scale, *BDI* Beck Depression Inventory, *WHOQOL-BREF* World Health Organization Quality of Life Instruments(26 item), *BRMS* Bech- Rafaelsen Melancholia scale, *Bf- S* The ZERSSEN Mood Scale.

*Benzodiazepines (etc. Clonazepam, Estazolam, Zolpidem) was permitted.

The included studies were published between 2000 and 2020. 68^23,25–30,32–55,57–93^ of the RCTs originated in China, 2 of the RCTs^24,31^ originated in the United States, 1^56^of the RCTs originated in German. 59 studies^25–30,32–54,57–59,61–71,74,75,77–85,88–90,92,93^ were published in Chinese, while 12 studies^23,24,31,55,56,60,72,73,76,86,87,91^, were in English. 50 RCTs^23,25–27,32–37,39–44,48,50–54,58,59,61–63,65,67–80,84–87,89,90,92,93^ were two-arm trials, and 21^24,28–31,38,45–47,49,55–57,60,64,66,81–83,88,91^ were three-arm trials. Treatment duration for acupuncture or related therapies ranged from 2 to 24 weeks.

### Network meta-analysis

#### Change in depression scores

**Figure 2:**
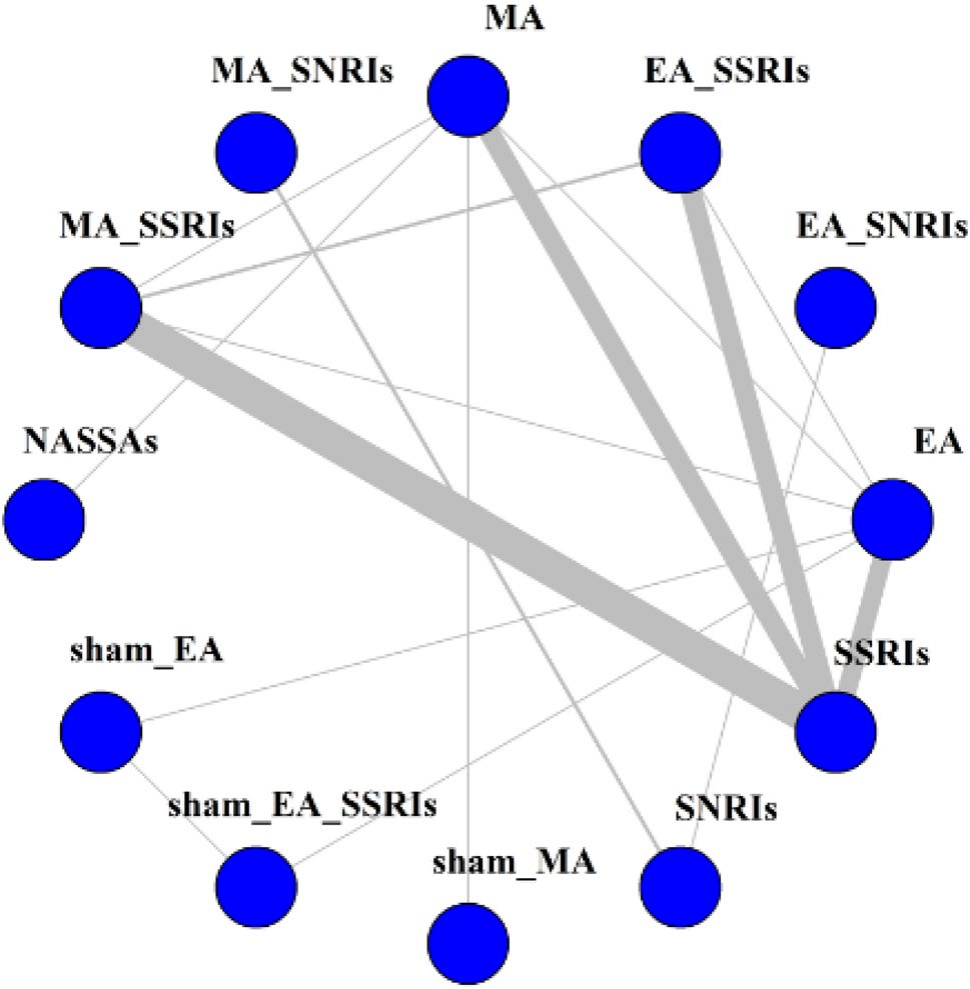
Network plot.

The network plot was presented in Fig. 2. Twelve interventions were involved: EA with SSRIs, MA with SSRIs, EA with SNRIs, MA with SNRIs, SNRIs, EA, MA, SSRIs, NASSAs, sham EA, sham MA, and sham EA with SSRIs. The two types of depression drugs, SSRIs and NASSAs, were included in this NMA. However, three therapies included the EA with SNRIs, MA with SNRIs, and SNRIs therapies were not able to form a connected loop with other interventions. Therefore, they were not be compared and analyzed in the main NMA.

**Figure 3:**
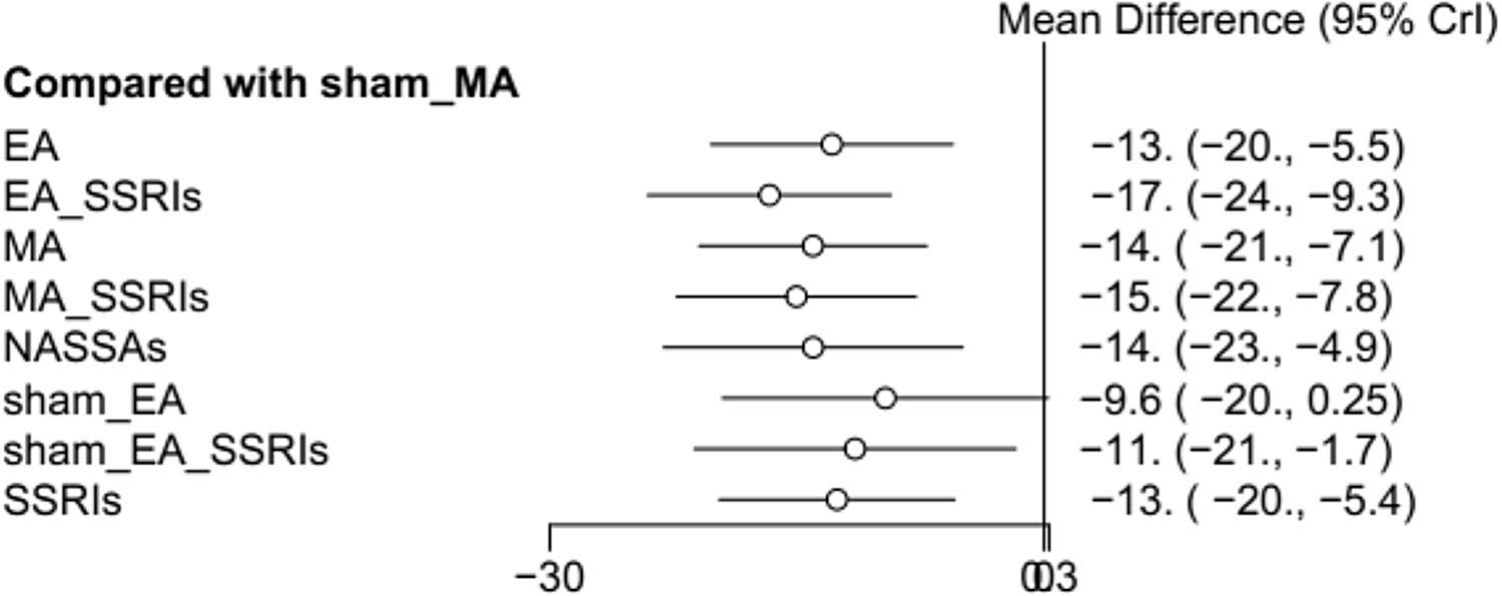
Forest plot compared with sham MA.

Fifty studies involving 3881 patients in main NMA reported changes in depression scores using the HAMD scale. Six three-arm-based studies and 44 two-arm-based studies were included. Among these studies, 19 studies (n = 19, 38.00%) were comparing MA plus SSRIs with SSRIs alone. And the rest were MA versus (vs) SSRIs (n = 11, 22.00%) and EA plus SSRIs vs SSRIs (n = 11, 22.00%), EA vs SSRIs (n = 10, 20.00%). The results of the NMA of different interventions were displayed in Table 2. For the combined interventions, the results of NMA indicated that EA with SSRIs was more effective in improving depression symptoms compared with MA, Sham EA, Sham MA, and SSRIs (MD: − 2.64, 95% CI: − 5.19 to − 0.10; MD: − 7.04, 95% CI: − 14.10 to − 0.03; MD: − 16.65, 95% CI: − 23.98 to − 9.34; MD: − 4.11, 95% CI: − 5.89 to − 2.33). And for MA with SSRIs, it seemed to be more effective as compared to SSRIs (MD: − 2.47, 95% CI: − 3.85 to − 1.11). For the acupuncture alone, MA was better than sham MA in reducing depression symptoms (MD: − 14.02, 95% CI: − 20.89, − 7.15). The EA could be more effective for relieving the depression symptoms compared with sham MA (MD: − 12.87, 95% CI: − 20.15 to − 5.56). Among all the interventions, EA with SSRIs seemed to achieve superior outcomes when compared to sham MA (MD: − 17.00, 95% CI: − 24.00 to − 9.30) (Fig. 3).

**Table 2:** Results of network meta-analysis for all possible treatment effects.

**EA**	**− 3.78 (− 6.26, − 1.33)**	− 1.14 (− 3.63, 1.32)	− 2.14 (− 4.37, 0.04)	− 1.11 (− 7.57, 5.27)	3.26 (− 3.32, 9.84)	1.43 (− 4.94, 7.8)	**12.87 (5.56, 20.15)**	0.33 (− 1.49, 2.12)
**3.78 (1.33, 6.26)**	**EA with SSRIs**	**2.64 (0.1, 5.19)**	1.63 (− 0.52, 3.79)	2.66 (− 3.81, 9.1)	**7.04 (0.03, 14.1)**	5.2 (− 1.63, 12.06)	**16.65 (9.34, 23.98)**	**4.11 (2.33, 5.89)**
1.14 (− 1.32, 3.63)	**− 2.64 (− 5.19, − 0.1)**	**MA**	− 1.01 (− 3.24, 1.22)	0.03 (− 5.9, 5.93)	4.4 (− 2.63, 11.45)	2.56 (− 4.27, 9.45)	**14.02 (7.15, 20.89)**	1.47 (− 0.37, 3.3)
2.14 (− 0.04, 4.37)	− 1.63 (− 3.79, 0.52)	1.01 (− 1.22, 3.24)	**MA with SSRIs**	1.03 (− 5.3, 7.36)	5.41 (− 1.52, 12.37)	3.57 (− 3.15, 10.36)	**15.02 (7.8, 22.25)**	**2.47 (1.11, 3.85)**
1.11 (− 5.27, 7.57)	− 2.66 (− 9.1, 3.81)	− 0.03 (− 5.93, 5.9)	− 1.03 (− 7.36, 5.3)	**NASSAs**	4.38 (− 4.79, 13.59)	2.54 (− 6.49, 11.63)	**14 (4.91, 23.07)**	1.44 (− 4.74, 7.64)
− 3.26 (− 9.84, 3.32)	**− 7.04 (− 14.1, − 0.03)**	− 4.4 (− 11.45, 2.63)	− 5.41 (− 12.37, 1.52)	− 4.38 (− 13.59, 4.79)	**Sham EA**	− 1.83 (− 8.51, 4.83)	9.62 (− 0.26, 19.46)	− 2.94 (− 9.78, 3.9)
− 1.43 (− 7.8, 4.94)	− 5.2 (− 12.06, 1.63)	− 2.56 (− 9.45, 4.27)	− 3.57 (− 10.36, 3.15)	− 2.54 (− 11.63, 6.49)	1.83 (− 4.83, 8.51)	**Sham EA with SSRIs**	**11.46 (1.74, 21.12)**	− 1.09 (− 7.75, 5.53)
**− 12.87 (− 20.15, − 5.56)**	**− 16.65 (− 23.98, − 9.34)**	**− 14.02 (− 20.89, − 7.15)**	**− 15.02 (− 22.25, − 7.8)**	**− 14 (− 23.07, − 4.91)**	− 9.62 (− 19.46, 0.26)	**− 11.46 (− 21.12, − 1.74)**	**Sham MA**	**− 12.54 (− 19.64, − 5.44)**
− 0.33 (− 2.12, 1.49)	**− 4.11 (− 5.89, − 2.33)**	− 1.47 (− 3.3, 0.37)	**− 2.47 (− 3.85, − 1.11)**	− 1.44 (− 7.64, 4.74)	2.94 (− 3.9, 9.78)	1.09 (− 5.53, 7.75)	12.54 (5.44, 19.64)	**SSRIs**

The estimates of mean difference of treatments in the columns versus rows presented in the lower diagonal elements (while those of the row treatments vs. column treatments are presented in the upper diagonal elements). Significant results are in bold and underscored.

*EA* electroacupuncture, *MA* manual acupuncture, *SSRIs* selective serotonin reuptake inhibitors, *NASSAs* noradrenaline and specific serotoninergic antidepressants, *TCAs* tricyclic antidepressants.

Table 3 presented the mean values of SUCRA, the hierarchy of eleven treatments on outcomes. According to SUCRA, EA plus SSRIs had the highest probability on improving depression symptoms with probabilities of 0.9518. The next was MA with SSRIs (0.784). The probability of MA was very close to NASSAs, and the mean values of SUCRA were 0.6421 and 0. 6162 respectively. And the probability of EA was 0.4648. The lowest was sham MA group with probabilities of 0.0052.

**Table 3:** Ranking probability of different interventions.

Rank	Treatments, SUCRA	Different treatments durations	
		Short-term (1 ≤ x ≤ 4 weeks)	Mid-term (4 < x ≤ 8 weeks)
1	EA with SSRIs, 0.9518	EA with SSRIs, 0.9104	EA with SSRIs, 0.9737
2	MA with SSRIs, 0.784	MA with SSRIs, 0.8589	MA with SSRIs, 0.8147
3	MA, 0.6421	EA, 0.4939	MA, 0.6329
4	NASSAs, 0.6162	MA, 0.463	NASSAs, 0.607
5	EA, 0.4648	NASSAs, 0.4592	SSRIs, 0.4576
6	SSRIs, 0.3961	Sham EA with SSRIs, 0.3579	EA, 0.4339
7	Sham EA with SSRIs, 0.3912	SSRIs, 0.2722	Sham EA with SSRIs, 0.3599
8	Sham EA, 0.2486	Sham EA, 0.1846	Sham EA, 0.2177
9	Sham MA, 0.0052		Sham MA, 0.0027

The separated NMA results of acupuncture with SNRIs showed that MA plus SNRIs had the highest probability on improving depression symptoms with probabilities of 0.8994, followed by EA plus SNRIs (0.3956) and SNRIs (0.205).

#### Inconsistency between direct and indirect comparisons

Assessment of inconsistency between direct and indirect comparisons using a node-splitting model showed that there were no inconsistencies among most studies (*P* > 0.05). The details of results were listed in Table 4.

**Table 4:** Node-splitting analysis of inconsistency.

Comparison	Direct RoM 95%CI	Indirect RoM 95%CI	Network RoM 95%CI	*P*-value
EA with SSRIs vs EA	− 5.2 (− 11.00, 0.94)	− 3.5 (− 6.30, − 0.75)	− 3.8 (− 6.20, − 1.30)	0.614
MA vs EA	1.8 (− 4.80, 8.50)	− 1.6 (− 4.30, 1.00)	− 1.2 (− 3.60, 1.30)	0.339
MA with SSRIs vs EA	8.3 (3.90, 13.00)	− 3.9 (− 5.70, − 2.00)	− 2.2 (− 4.40, 0.06)	0.000
SSRIs vs EA	− 0.47 (− 2.00, 1.00)	− 3.6 (− 2.10, 9.20)	0.33(− 1.5, 2.10)	0.176
MA with SSRIs vs EA with SSRIs	2.2 (− 2.30, 6.80)	1.40 (− 1.10, 4.00)	1.6 (− 0.52, 3.80)	0.760
MA with SSRIs vs MA	− 3.7 (− 9.90, 2.50)	− 0.57 (− 3.00, 1.90)	− 1.00 (− 3.20, 1.20)	0.351
SSRIs vs MA	1.8 (− 0.10, 3.80)	− 1.8 (− 8.70, 5.20)	1.5 (− 0.37, 3.30)	0.322

*RoM* ratio of mean.

#### Subgroup analysis

The change in depression scores at the short-term (1 ≤ x ≤ 4 weeks) was reported among 40 studies, 41 studies reporting the change in depression scores at the mid-term (4 < x ≤ 8 weeks), six studies reporting the change in depression scores at the long-term (x > 8 weeks). The data of different interventions were analyzed according to the different treatment duration. For the short-term, there were eight different interventions. The treatment of EA with SSRIs had the largest probability of being the top rank intervention (0.9014), followed by MA with SSRIs (0.8589), EA (0.4939), MA (0.4630), and NASSAs (0.4592). For the mid-term, the highest probability on improving depression symptoms was EA with SSRIs similarly, with the probability of 0.9737. MA with SSRIs, MA, and NASSAs followed closely with probabilities of 0.8147, 0.6329, and 0.6070, respectively. For the long-term, six studies with four treatments (EA, SSRIs, MA with SNRIs, and SNRIs) were included. However, their network was disconnected.

Fourteen studies^34,35,38,42,43,49,52,53,55,61,70,73,76,91^ reported the change scores using the SDS. Besides, 3 studies^30,43,73^ used the Montgomery-Asberg Depression Rating Scale (MADRS). The corresponding network analysis failed to be conducted due to the limited number of studies.

### Adverse events

Twenty-four reported the presence of adverse events^24,25,28,36,39,41,43,50,53,55,58,59,61,62,74–76,78,80,84,85,87,89,91^. Among the acupuncture groups and control groups, the main comparable adverse reactions found were needle-related pain (6 cases)^24,76^ and skin erythema of acupoints (2 cases)^28,87^. These symptoms were slight and persisted for less than 2 days. One of the included studies reported that MA with SSRIs and EA with SSRIs groups had significantly fewer side effects as compared with the SSRIs group^91^. One serious adverse event was reported requiring hospitalization due to abnormal behaviors and confusion of mind in the MA with SSRIs group^91^. Due to a limited number of studies that reported the same adverse outcome, it was not analyzed using NMA.

### Quality of evidence

**Figure 4:**
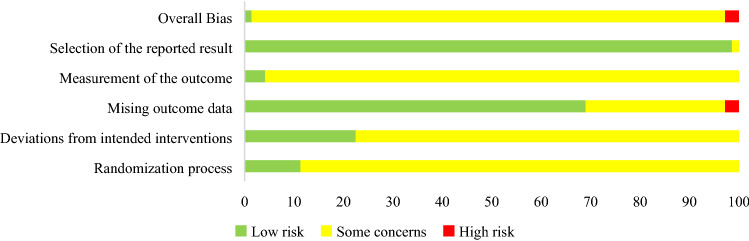
Risk of bias summery for 66 included studies.

Figure 4 and Table 5 presented the assessment results of the risks of bias. Most RCTs had a low risk of bias in selection of the reported result (n = 70, 99%) and missing outcome data (n = 49, 69%). However, a high proportion had concerns of bias in reporting measurement of the outcome (n = 68, 96%), randomization process (n = 63, 89%), and deviations from the intended interventions (n = 55, 77%). Regarding reports of interventions specified to acupuncture, STRICTA showed that majority of the RCTs reported details of needling (n = 71, 100%), details of other interventions administered to the acupuncture group (n = 47, 66%), instructions to practitioners, and information and explanations to patients (n = 40, 56%), and precise description of the control or comparator (n = 68, 96%). However, many RCTs did not report the descriptions of participating acupuncturists (n = 58, 82%), nor rationale for the control or comparator (n = 46, 65%). The details of the appraisal of acupuncture procedure based on STRICTA were presented in Table 6.

**Table 5:** Risk of bias assessment for 66 included studies.

Source		Randomization process	Deviations from intended interventions	Missing outcome data	Measurement of the outcome	Selection of the reported result	Overall
Ai et al.^23^	2018	S	L	L	S	L	S
Allen et al.^24^	2006	S	L	L	L	L	S
Chen et al.^25^	2014	S	L	L	S	L	S
Chen et al.^26^	2010	S	S	L	S	L	S
Chen et al.^27^	2011	S	S	S	S	L	S
Dong et al.^28^	2017	S	S	L	S	L	S
Duan et al.^29^	2008	S	S	L	S	L	S
Feng et al.^30^	2015	S	S	S	S	L	S
Gallagher et al.^31^	2001	S	L	H	S	L	H
Gu et al.^32^	2015	L	S	S	S	L	S
Guo et al^33^	2019	S	S	L	S	L	S
Han et al^34^	2019	L	S	L	S	L	S
Huang et al.^35^	2013	S	S	L	S	L	S
Jiang et al.^36^	2008	S	S	L	S	L	S
Li et al.^37^	2013	S	S	S	S	L	S
Li et al.^38^	2004	S	S	L	S	L	S
Lin et al.^39^	2004	S	S	L	S	L	S
Li et al.^40^	2017	S	S	S	S	L	S
Lin et al.^41^	2005	S	S	L	S	L	S
Lin et al.^42^	2014	S	S	S	S	L	S
Liu et al.^43^	2018	S	S	S	S	L	S
Liu et al.^44^	2005	S	S	L	S	L	S
Liu et al.^45^	2014	S	S	L	S	L	S
Liu et al.^46^	2015	S	S	L	S	L	S
Liu et al.^47^	2017	S	S	S	S	L	S
Lu et al.^48^	2017	S	S	S	S	L	S
Luo et al.^49^	2003	S	L	S	S	L	S
Ma et al.^50^	2011	L	L	L	S	L	S
Ma et al.^51^	2011	S	L	L	S	L	S
Ma et al.^52^	2011	S	S	L	S	L	S
Ma et al^53^	2020	S	S	L	S	L	S
Pei et al.^54^	2006	S	S	L	S	L	S
Qu et al.^55^	2013	L	L	L	S	L	S
Roschke et al.^56^	2000	S	L	S	S	L	S
Shi et al.^57^	2015	L	L	L	S	L	S
Song et al.^58^	2013	S	L	L	S	L	S
Sun et al.^59^	2012	S	S	L	S	L	S
Sun et al.^60^	2013	L	L	L	S	L	S
Tang et al.^61^	2003	S	S	L	S	L	S
Tian et al.^62^	2008	S	S	L	S	L	S
Wang et al.^63^	2018	S	S	S	S	L	S
Wang et al.^64^	2014	S	S	L	S	L	S
Wang et al.^65^	2016	S	L	L	S	L	S
Wang et al.^66^	2007	S	S	S	S	L	S
Wang et al.^67^	2007	S	S	S	S	L	S
Wang et al.^68^	2006	S	S	L	S	L	S
Wang et al.^69^	2007	S	S	S	S	L	S
Wang et al.^70^	2008	S	S	L	S	L	S
Wang et al.^71^	2010	S	S	L	S	L	S
Wang et al.^72^	2014	S	S	L	S	L	S
Wang et al.^73^	2017	S	L	L	L	L	S
Wang et al^74^	2020	S	S	L	S	L	S
Wang et al^75^	2019	S	S	L	S	L	S
Wang et al.^76^	2013	S	L	L	S	L	S
Wen et al.^77^	2003	S	S	L	S	L	S
Wu et al.^78^	2010	S	S	L	S	L	S
Xu et al.^79^	2009	S	S	S	S	L	S
Xu et al.^80^	2004	S	S	L	S	L	S
Xu et al.^81^	2011	S	S	L	S	L	S
Yang et al.^82^	2012	S	S	S	S	L	S
Yi et al.^83^	2011	S	S	L	S	L	S
Zhang et al.^84^	2007	S	S	S	S	L	S
Zhang et al.^85^	2012	S	S	L	S	L	S
Zhang et al.^86^	2007	S	S	H	S	S	H
Zhang et al.^87^	2009	L	L	L	L	L	L
Zhao et al.^88^	2010	S	S	L	S	L	S
Zhao et al.^89^	2010	S	S	S	S	L	S
Zhao et al.^90^	2006	S	S	S	S	L	S
Zhao et al.^91^	2019	L	S	L	S	L	S
Zheng et al.^92^	2012	S	S	L	S	L	S
Zhu et al.^93^	2018	S	S	L	S	L	S

*L* low risk, *S* some concerns, *H* high risk.

**Table 6:** Appraisal of acupuncture procedure based on STRICTA.

Source		1a	1b	1c	2a	2b	2c	2d	2e	2f	2g	3a	3b	4a	4b	5	6a	6b
Ai et al.^23^	2018	TCM	Y	Y	N	N	Y	N	Y	Y	Y	Y	Y	Y	Y	N	N	Y
Allen et al.^24^	2006	TCM	Y	Y	Y	Y	Y	Y	Y	Y	Y	Y	Y	Y	Y	Y	Y	Y
Chen et al.^25^	2014	TCM	Y	Y	N	N	Y	N	Y	Y	N	Y	Y	Y	Y	N	Y	Y
Chen et al.^26^	2010	TCM	Y	Y	N	Y	Y	Y	Y	Y	Y	Y	Y	N	N	N	N	Y
Chen et al.^27^	2011	TCM	Y	Y	N	N	N	Y	Y	Y	N	Y	Y	Y	N	N	N	Y
Dong et al.^28^	2017	TCM	Y	Y	N	Y	Y	Y	Y	Y	Y	Y	Y	Y	N	N	N	Y
Duan et al.^29^	2008	TCM	Y	Y	N	N	N	N	Y	N	N	Y	Y	N	Y	N	N	Y
Feng et al.^30^	2015	TCM	Y	Y	N	Y	N	Y	Y	Y	Y	Y	Y	Y	Y	N	N	Y
Gallagher et al.^31^	2001	TCM	Y	Y	N	Y	N	N	Y	N	N	Y	N	N	N	N	Y	Y
Gu et al.^32^	2015	TCM	Y	Y	N	Y	Y	Y	Y	Y	Y	Y	Y	N	Y	N	N	Y
Guo et al^33^	2019	TCM	Y	Y	N	Y	Y	Y	Y	Y	Y	Y	Y	N	Y	N	Y	Y
Han et al^34^	2019	TCM	Y	Y	N	Y	Y	N	Y	Y	Y	Y	Y	N	Y	N	Y	Y
Huang et al.^35^	2013	TCM	Y	Y	N	Y	Y	N	Y	Y	Y	Y	Y	Y	Y	N	Y	Y
Jiang et al.^36^	2008	TCM	Y	Y	N	N	N	Y	Y	Y	N	Y	Y	Y	Y	N	N	Y
Li et al.^37^	2013	TCM	Y	Y	N	Y	N	Y	Y	Y	Y	Y	Y	Y	N	N	N	Y
Li et al.^38^	2004	TCM	Y	Y	N	N	Y	N	Y	Y	N	Y	Y	N	N	N	N	Y
Lin et al.^39^	2004	TCM	Y	Y	N	N	Y	N	N	N	N	Y	Y	Y	N	N	N	Y
Li et al.^40^	2017	TCM	Y	Y	N	Y	Y	N	Y	Y	Y	Y	Y	Y	N	N	N	Y
Lin et al.^41^	2005	TCM	Y	Y	N	N	N	N	N	N	N	Y	Y	Y	Y	N	N	Y
Lin et al.^42^	2014	TCM	Y	Y	N	N	N	Y	Y	Y	N	Y	Y	Y	N	N	N	Y
Liu et al.^43^	2018	TCM	Y	Y	N	Y	Y	N	Y	Y	Y	Y	Y	Y	Y	N	Y	Y
Liu et al.^44^	2005	TCM	Y	Y	N	N	N	Y	Y	N	N	Y	N	Y	N	N	Y	Y
Liu et al.^45^	2014	TCM	Y	Y	N	Y	N	N	Y	Y	Y	Y	Y	Y	Y	N	N	Y
Liu et al.^46^	2015	TCM	Y	Y	N	Y	Y	N	Y	Y	N	Y	Y	Y	Y	N	N	Y
Liu et al.^47^	2017	TCM	Y	Y	N	Y	Y	Y	Y	Y	Y	Y	Y	Y	Y	N	N	Y
Lu et al.^48^	2017	TCM	Y	Y	N	Y	Y	N	Y	Y	Y	Y	Y	Y	N	N	N	Y
Luo et al.^49^	2003	TCM	Y	Y	N	Y	Y	N	Y	N	Y	Y	Y	N	Y	Y	Y	Y
Ma et al.^50^	2011	TCM	Y	Y	N	Y	Y	Y	Y	Y	Y	Y	Y	N	Y	N	Y	Y
Ma et al.^51^	2011	TCM	Y	Y	N	N	Y	Y	Y	Y	Y	Y	Y	Y	Y	N	Y	Y
Ma et al.^52^	2011	TCM	Y	Y	N	N	Y	Y	Y	Y	N	Y	Y	Y	Y	N	Y	Y
Ma et al^53^	2020	TCM	Y	Y	N	Y	Y	N	Y	Y	Y	Y	Y	N	Y	Y	Y	Y
Pei et al.^54^	2006	TCM	Y	Y	N	Y	Y	Y	Y	Y	Y	Y	Y	N	N	N	N	N
Qu et al.^55^	2013	TCM	Y	Y	N	Y	Y	Y	Y	Y	Y	Y	Y	Y	Y	Y	Y	Y
Roschke et al.^56^	2000	TCM	Y	Y	N	Y	Y	N	Y	Y	Y	Y	Y	Y	Y	Y	N	Y
Shi et al.^57^	2015	TCM	Y	Y	N	Y	Y	Y	Y	Y	Y	Y	Y	N	Y	N	N	Y
Song et al.^58^	2013	TCM	Y	Y	N	N	Y	Y	Y	Y	N	Y	Y	N	Y	N	Y	Y
Sun et al.^59^	2012	TCM	Y	Y	N	N	N	N	Y	Y	N	Y	Y	Y	N	N	Y	Y
Sun et al.^60^	2013	TCM	Y	Y	N	Y	Y	Y	Y	Y	Y	Y	Y	N	N	Y	Y	Y
Tang et al.^61^	2003	TCM	Y	Y	N	N	N	N	Y	Y	N	Y	Y	Y	N	N	N	Y
Tian et al.^62^	2008	TCM	Y	N	N	N	N	Y	Y	Y	N	Y	Y	Y	Y	N	N	Y
Wang et al.^63^	2018	TCM	Y	Y	N	N	N	N	Y	Y	N	Y	Y	Y	N	Y	N	Y
Wang et al.^64^	2014	TCM	Y	Y	N	Y	N	Y	Y	Y	N	Y	Y	Y	Y	Y	Y	Y
Wang et al.^65^	2016	TCM	Y	Y	N	Y	Y	N	Y	Y	Y	Y	Y	N	Y	N	N	Y
Wang et al.^66^	2007	TCM	Y	Y	N	Y	Y	Y	Y	Y	Y	Y	Y	N	N	N	N	Y
Wang et al.^67^	2007	TCM	Y	Y	N	Y	N	Y	Y	Y	Y	Y	Y	Y	N	N	N	Y
Wang et al.^68^	2006	TCM	Y	Y	N	Y	N	Y	Y	Y	N	Y	Y	Y	Y	N	N	Y
Wang et al.^69^	2007	TCM	Y	Y	N	Y	N	N	Y	Y	Y	Y	Y	N	N	N	N	Y
Wang et al.^70^	2008	TCM	Y	Y	N	Y	Y	Y	Y	Y	Y	Y	Y	Y	N	N	N	Y
Wang et al.^71^	2010	TCM	Y	Y	N	Y	Y	Y	Y	Y	Y	Y	Y	N	Y	N	N	Y
Wang et al.^72^	2014	TCM	Y	Y	Y	Y	Y	Y	Y	Y	Y	Y	Y	Y	N	Y	Y	Y
Wang et al.^73^	2017	TCM	Y	Y	Y	Y	Y	Y	Y	Y	Y	Y	Y	Y	N	Y	Y	Y
Wang et al^74^	2020	TCM	Y	Y	N	Y	Y	Y	Y	Y	Y	Y	Y	Y	Y	N	Y	Y
Wang et al^75^	2019	TCM	Y	Y	N	Y	Y	Y	Y	Y	Y	Y	Y	N	Y	N	N	Y
Wang et al.^76^	2013	TCM	Y	Y	Y	Y	Y	Y	Y	Y	Y	Y	Y	N	N	Y	N	Y
Wen et al.^77^	2003	TCM	Y	N	N	N	N	Y	Y	Y	N	Y	Y	N	N	N	N	N
Wu et al.^78^	2010	TCM	Y	Y	N	N	N	Y	Y	Y	N	Y	Y	Y	Y	N	N	Y
Xu et al.^79^	2009	TCM	Y	Y	N	N	Y	N	Y	Y	Y	Y	Y	N	N	N	N	Y
Xu et al.^80^	2004	TCM	Y	Y	N	Y	N	N	N	Y	Y	Y	Y	Y	N	N	N	Y
Xu et al.^81^	2011	TCM	Y	Y	N	Y	Y	Y	N	Y	Y	Y	Y	Y	N	N	N	Y
Yang et al.^82^	2012	TCM	Y	Y	N	N	N	Y	Y	Y	N	Y	Y	Y	Y	N	N	Y
Yi et al.^83^	2011	TCM	Y	Y	N	Y	Y	Y	Y	Y	Y	Y	Y	Y	Y	N	N	Y
Zhang et al.^84^	2007	TCM	Y	Y	N	N	N	N	Y	Y	N	Y	Y	Y	Y	N	Y	N
Zhang et al.^85^	2012	TCM	Y	Y	N	Y	Y	N	Y	Y	Y	Y	Y	N	N	N	N	Y
Zhang et al.^86^	2007	TCM	Y	Y	N	Y	N	N	Y	N	N	Y	Y	Y	N	N	Y	Y
Zhang et al.^87^	2009	TCM	Y	Y	Y	Y	Y	Y	Y	Y	Y	Y	Y	Y	Y	Y	Y	Y
Zhao et al.^88^	2010	TCM	Y	Y	N	Y	N	Y	Y	Y	Y	Y	Y	Y	Y	N	N	Y
Zhao et al.^89^	2010	TCM	Y	Y	N	N	Y	Y	Y	Y	N	Y	Y	Y	N	N	N	Y
Zhao et al.^90^	2006	TCM	Y	Y	N	Y	N	N	Y	Y	Y	Y	Y	N	N	N	N	Y
Zhao et al.^91^	2019	TCM	Y	Y	N	Y	Y	Y	Y	Y	Y	Y	Y	Y	Y	Y	Y	Y
Zheng et al.^92^	2012	TCM	Y	Y	N	Y	Y	N	Y	Y	Y	Y	Y	Y	Y	N	N	Y
Zhu et al.^93^	2018	TCM	Y	Y	N	Y	N	N	Y	Y	Y	Y	Y	Y	Y	N	N	Y

(1a) Style of acupuncture; (1b) Reasoning for treatment provided; (1c) Extent to which treatment was varied; (2a) Number of needle insertions per subject per session; (2b) Names of points used; (2c) Depth of insertion; (2d) Response sought; (2e) Needle stimulation; (2f) Needle retention time; (2g) Needle type; (3a) Number of treatment sessions; (3b) Frequency and duration of treatment sessions; (4a) Details of other interventions administered to the acupuncture group; (4b) Setting and context of treatment, including instructions to practitioners, and information and explanations to patients; (5) Description of participating acupuncturists; (6a) Rationale for the control or comparator in the context of the research question, with sources that justify this choice; (6b) Precise description of the control or comparator; Y: Reported; N: not available.

### Summary of findings GRADE

The summary of quality of evidence of change in depression scores between comparisons was presented in Table 7. Because of high risk of bias, imprecise confidence interval, and inconsistency, almost all comparisons for the reduction of depression proved low quality evidence except for the comparison of EA with SSRIs vs EA (moderate quality evidence), which indicated that most comparisons might result in little or no difference in reducing depression scores.

**Table 7:** Summary of findings’ table of comparisons in change in depression scores.

Comparison	Direct estimates 95%CI	Certainty of evidence	Indirect estimates 95%CI	Certainty of evidence	Network estimates 95%CI	Certainty of evidence
EA with SSRIs vs EA	− 5.2 (− 11.00, 0.94)	Low ^a,b^	− 3.5 (− 6.30, − 0.75)	Moderate ^a^	− 3.8 (− 6.20, − 1.30)	Moderate ^a^
MA vs EA	1.8 (− 4.80, 8.50)	Low ^a,d^	− 1.6 (− 4.30, 1.00)	Low ^a,b^	− 1.14 (− 3.63, 1.32)	Very low ^a,b,c,d^
MA with SSRIs vs EA	8.3 (3.90, 13.00)	Low ^a,d^	− 3.9 (− 5.70, − 2.00)	Low ^a,b^	− 2.14 (− 4.37, 0.04)	Very low ^a,b,d,e^
SSRIs vs EA	− 0.47 (− 2.00, 1.00)	Low ^a,b^	− 3.6 (− 2.10, 9.20)	Low ^a,b^	0.33 (− 1.49, 2.12)	Low ^a,b^
MA with SSRIs vs EA with SSRIs	2.2 (− 2.30, 6.80)	Low ^a,b^	1.40 (− 1.10, 4.00)	Low ^a,b^	1.63 (− 0.52, 3.79)	Low ^a,b^
MA with SSRIs vs MA	− 3.7 (− 9.90, 2.50)	Low ^a,b^	− 0.57 (− 3.00, 1.90)	Low ^a,b^	− 1.01 (− 3.24, 1.22)	Low ^a,b^
SSRIs vs MA	1.8 (− 0.10, 3.80)	Low ^a,b^	− 1.8 (− 8.70, 5.20)	Low ^a,b^	1.47 (− 0.37, 3.3)	Low ^a,b^

^a^Downgrading for risk of bias; ^b^downgrading for imprecision (wide confidence interval); ^c^downgrading for incoherence; ^d^downgrading for inconsistency; ^e^downgrading for intransitivity.

## Discussion

### Main results

To our knowledge, this study is the first NMA that explored the efficiency of different techniques of acupuncture comparing with common pharmacological treatments or other non-medication therapies for MDD. Comparing with the most updated meta-analyses focused on the effect of acupuncture on MDD^94,95^, NMA allows ranking of all different treatment options through the quantitative comparison of interventions from a comprehensive collection of literature. The pooled results showed that the combined interventions (EA with SSRIs, and MA with SSRIs) obtained a better efficacy for improving depression symptoms compared to acupuncture, pharmacological interventions alone, or other inactive groups. Even the studies observing SNRIs and SNRIs combined with EA or MA were not analyzed in main NMA, add-on therapies were more effective than pharmacological interventions alone. Among all the regimens, EA with SSRIs had the highest probability on improving depression symptoms, while the estimation of MA with SSRIs was very close to EA with SSRIs. Besides, for different treatment durations, there were slight differences. For the short-term (1 ≤ x ≤ 4 weeks) and mid-term (4 < x ≤ 8 weeks), both EA with SSRIs and MA with SSRIs achieved better efficacy. However, EA was more effective than MA for the short-term, while the situation reversed for the mid-term.

Based on the comparison of adverse effects among the groups from all included studies, acupuncture alone and its combinations were proved to be relatively safe therapies for MDD patients. Although one case^91^ of serious adverse effect was reported, no direct association between the intervention and the case was justified.

Considerable experimental and clinical evidence suggest that MDD is a neuro-endocrine-immune system disorder, and more novel mechanisms are explored basing on new genetic, epigenetic and optogenetic tools^96^. The exact mechanism why EA with SSRIs shows the best treatment efficiency for MDD patients is still not fully understood. According to early animal electrophysiological and immunohistochemical studies, EA can modify the activities of serotonergic neurons in the dorsal raphe (DR) and raphe magnus (RMg), activate serotonin- and catecholamine-containing neurons in the RMg and locus coeruleus^97,98^. In the clinical study, EA can restore the normal concentration of glial cell-derived neurotrophic factor (GDNF) in the serum of MDD patients which having similar effect to fluoxetine^60^. Furthermore, EA combined with SSRIs can increase serum 5-HT more rapidly, reduce pro-inflammatory cytokines secreted by TH1 cells, and increase anti-inflammatory cytokines secreted by TH2 cells^99^. Further studies are required to answer whether these observations are based on the simple add-on effects, or due to more complex vivo interaction pathways.

### Implications for practice

The comparisons among various treatment approaches provided updated evidence for practitioners in the areas of CAM and integrative medicine and decision-makers in deriving public health policies. The results in the subgroup analysis indicated that acupuncture with common pharmacological treatments or acupuncture alone could be more effective for MDD even in a short treatment cycle. Under the synthesis of data, we suggest that acupuncture with common pharmacological treatments could be considered as better therapeutic approaches.

Nowadays, with the development of the registration system of acupuncturists and the increasing popularity of acupuncture services worldwide^100^, acupuncture could be a practical option for MDD patients. In the current clinical practice guideline developed by the American College of Physicians Clinical Guidelines Committee, acupuncture has been studied as a potential monotherapy and combination therapy with antidepressants on treating patients with MDD^101^. However, the citation of acupuncture articles is limited in the guideline. Although acupuncture trials are largely conducted and published on Chinese databases, the evidence from Chinese databases is largely skipped in the guideline. In this NMA, clinical trial data in recent years from Chinese databases was included. Results of this study provided significance evidence-based data by systematically estimating the clinical effect and safety of acupuncture and its combinations.

### Limitation

This study had several limitations: (i) although various outcome measures were collected, only HAMD was included in NMA because of insufficient data from the other scales; (ii) included studies were mainly carried out in Chinese populations; (iii) incomplete reporting of trial details might have affected the reliability of results; (iv) only 9 types of interventions were analyzed for the main network analysis. We intended to involve more non-medication therapies. However, after systematic searching, we only found one study which explored the effect of cognitive-behavior therapy for MDD. Given the limited study data, it was not included in the NMA. Therefore, more studies focusing on non-medication interventions would be needed.

Authors of the RCTs included in this review could have improved their publications by reporting the details of randomization process and measurement of the outcomes, increasing the data transparency through demonstrating the design and every afford involved in the clinical study. Moreover, be specific to acupuncture-related trials, authors are encouraged to report the qualification or years in acupuncture practice for acupuncturists participated in the trials, and to provide justification for the choice of the control or comparator in the context of the research question.

Indeed, the lack of long-term follow-up studies made it difficult to achieve more profound research significance. Patients with MDD often suffer from longer disease cycles and high recurrence rates. We need more evidence to prove that acupuncture not only could show improvements on the depression rating scales, but also more benefits such as drug truncation, low recurrence rate, shorter treatment cycle.

## Conclusion

Acupuncture and its combinations could be safe and effective interventions for MDD patients. What’s more, EA with SSRIs seems to be the most effective intervention among the assessed interventions. Well-designed and large-scale studies with long-term follow-up should be conducted in the future.

## Supplementary Information

Supplementary Information.

## Data Availability

The data that support the findings of this study are available from the corresponding author upon reasonable request.

## Abbreviations

MDD: Major depressive disorder
EA: Electro-acupuncture
SSRIs: Selective serotonin reuptake inhibitors
MA: Manual acupuncture
SNRIs: Serotonin-norepinephrine reuptake inhibitors
TCAs: Tricyclic antidepressants
US: United States
SGA: Second generation antidepressant
CAM: Complementary and alternative medicine
NMA: Network meta-analysis
AMED: Allied and complementary medicine database
CNKI: China national knowledge infrastructure
CBM, CBMdisc: China biology medicine disc
CQVIP: Chongqing VIP database
PRISMA: Preferred reporting items for systematic reviews and meta-analyses
PRISMA-NMA: PRISMA extension statement for reporting of systematic reviews incorporating network meta-analyses of health care interventions
RCTs: Randomized control trials
DSM: The diagnostic and statistical manual of mental disorders
ICD: The international classification of diseases
CCMD: The Chinese classification of mental disorders
HDRS (also abbreviated as HAMD): Hamilton Depression Rating Scale
SDS: The Self-Rated Depression Scale
SERS: Side Effect Rating Scale
TESS: Treatment Emergent Symptom Scale
STRICTA: The revised standards for reporting interventions in clinical trials of acupuncture
GRADE: The grading of recommendations assessment, development and evaluation
MDs: Mean differences
SD: Standard deviation
CI: Confidence interval
SUCRA: The cumulative ranking curve
MAOIs: Monoamine oxidase inhibitors
NASSAs: Noradrenaline and specific serotoninergic antidepressants
MADRS: The Montgomery-Asberg Depression Rating Scale
