# Supplementary material for: A network meta-analysis on the effectiveness and safety of acupuncture in treating patients with major depressive disorder
Source: Sci Rep. 2021 May 17;11:10384. doi: 10.1038/s41598-021-88263-y (PMC8129113; doi:10.1038/s41598-021-88263-y)
Supplement: Supplementary file 1 — Supplementary Information. [file 41598_2021_88263_MOESM1_ESM.docx]

**Supplementary Information**

**Title:** A Network Meta-Analysis on the Effectiveness and Safety of Acupuncture in Treating Patients with Major Depressive Disorder

**Author list:** Hu Zhichao; Lam Wai Ching; Li Huijuan; Yao Liang; Wang Zhiyu; Huang Weiyang; Bian Zhaoxiang; Zhong LD Linda

**Supplementary Methods. Search Strategy**

**1. PubMed**#1 “Acupuncture”[Mesh]

#2 “Acupuncture Therapy”[Mesh]

#3 “Electroacupuncture”[Mesh]

#4 Acupuncture  [tiab]

#5 Acupressure [tiab]

#6 acupunctur* [tiab]

#7 acupoint* [tiab]

#8 needling [tiab]

#9 electrostimulation[tiab]

#10 auriculoacupuncture[tiab]

#11 Electro-acupuncture [tiab]

#12 Electroacupuncture [tiab]

#13 OR #1-12

#14 “Depressive Disorder, Major” [MeSH]

#15 major depressive disorde r [tiab]

#16 major depression [tiab]

#17 OR #14-16

#18 “Randomized Controlled Trial”[Publication Type] OR “Randomized Controlled Trials as Topic”[MeSH] OR “Randomized Controlled Trial”[tiab] OR “Single-Blind Method”[MeSH] OR “Double-Blind Method”[MeSH] OR “Random Allocation”[MeSH]

#19 “Controlled Clinical Trial”[pt] OR “Controlled Clinical Trials as Topic”[Mesh] OR controlled clinical trial*[tiab] OR controlled trial*[tiab] OR controlled stud*[tiab]

#20 OR #18-19

#21 “Animals”[Mesh] NOT “Humans”[Mesh])

#22 #20 NOT #21

#23 #13 AND #17 AND #22 AND 1990:2021[dp] AND (english[la])

**2. Embase and AMED (via ovid)**

1 exp major depressive disorder /

2 major depressive disorder.mp.

3 exp major depression /

4 major depression.mp.

5 Or/1-4

6 exp Acupuncture /

7 exp Acupuncture Therapy

8 exp Electroacupuncture

9 Acupuncture

10 Acupressure

11 acupunctur*

12 acupoint*

13 needling

14 electrostimulation

15 auriculoacupuncture

16 Electro-acupuncture

17 Electroacupuncture

18 or/6-17

19 random$.tw.

20 factorial$.tw.

21 (crossover$ or cross over$ or cross-over$).tw.

22 placebo$.tw.

23 single blind.mp.

24 double blind.mp.

25 triple blind.mp.

26 (singl$ adj blind$).tw.

27 (double$ adj blind$).tw.

28 (tripl$ adj blind$).tw.

29 assign$.tw.

30 allocat$.tw.

31 crossover procedure/

32 double blind procedure/

33 single blind procedure/

34 triple blind procedure/

35 randomized controlled trial/

36 or/19-35

37 animal.sh.

38 human,sh.

39 37 not 38

40 36 not 39

41 5 and 18 and 40

[mp=title, abstract, subject headings, heading word, drug trade name, original title, device manufacturer, drug manufacturer, device trade name, keyword]

**3.Cochrane library**

1 MeSH descriptor: [Depressive Disorder, Major] explode all trees

2 major depressive disorder:ti,ab,kw

3 major depression:ti,ab,kw

4 or/1-3

5 MeSH descriptor: [Acupuncture Therapy] explode all trees

6 MeSH descriptor: [Acupuncture] explode all trees

7 MeSH descriptor: [Electroacupuncture] explode all trees

8 Acupuncture:ti,ab,kw

9 Acupressure

10 acupunctur*

11 acupoint*

12 needling

13 electrostimulation

14 auriculoacupuncture

15 Electro-acupuncture

16 Electroacupuncture

17 or/5-16

18 4 and 17

**4. CNKI/ CBM/ Wan Fang Data/ CNVIP**

The Chinese version available on request from reviewers or readers.
